# Inhaled Cannabis, Asthma, and Chronic Obstructive Pulmonary Disease: A Population-Based Cross-Sectional Study of *n* = 379,049

**DOI:** 10.1007/s11606-025-09833-8

**Published:** 2025-09-04

**Authors:** Alison S. Rustagi, Abra M. Jeffers, F. Julian Graham, Beth E. Cohen, Christopher G. Slatore, Amy L. Byers, Stanton A. Glantz, Salomeh Keyhani

**Affiliations:** 1 Center for Data to Discovery and Delivery Innovation (3DI), San Francisco VA Health Care System, San Francisco, CA USA; 2 Division of General Internal Medicine, University of California-San Francisco, San Francisco, CA USA; 3 Mongan Institute Health Policy Research Center, Massachusetts General Hospital, Boston, MA USA; 4 Division of General Academic Pediatrics, Massachusetts General Hospital for Children, Boston, MA USA; 5 Tobacco Research and Treatment Center, Massachusetts General Hospital, Boston, MA USA; 6 Center to Improve Veteran Involvement in Care, VA Portland Health Care System, Portland, OR USA; 7 National Center for Lung Cancer Screening, Veterans Health Administration, Washington, DC USA; 8 Division of Pulmonary, Critical Care, and Allergy Medicine, Oregon Health & Science University, Portland, OR USA; 9 Research Service, San Francisco Veterans Affairs Health Care System, San Francisco, CA USA; 10 Department of Psychiatry and Behavioral Sciences, University of California, San Francisco, CA USA; 11 Retired, Center for Tobacco Control Research & Education and Department of Medicine, University of California, San Francisco, CA USA; 12 Weill Institute for Neurosciences, University of California, San Francisco, CA USA; 13 Northern California Institute for Research and Education, San Francisco, CA USA

**Keywords:** asthma, cannabis, COPD, marijuana

## Abstract

**Background:**

Cannabis may cause chronic pulmonary disease. Prior studies have been limited by low cannabis exposure, lack of data on tobacco cigarettes, and/or limited numbers of those without tobacco cigarette use.

**Objective:**

To examine whether inhaled cannabis associated with asthma and chronic obstructive pulmonary disease, independent of tobacco cigarettes.

**Design:**

Cross-sectional analysis of population-based, nationally representative survey data.

**Participants:**

Adults 18–74 years who participated in the 2016–2020 Behavioral Risk Factor Surveillance System surveys.

**Main Measures:**

The exposure was past-30-day cannabis use, from 0 (0/30 days) to 1 (30/30 days). Outcomes were self-reported diagnoses by a medical professional of asthma or chronic obstructive pulmonary disease. We used multivariable logistic regression to test whether inhaled cannabis was associated with odds of disease, adjusted for sociodemographics and tobacco cigarette use (current/former/never). Pre-specified analyses restricted to those with no lifetime tobacco cigarette use.

**Key Results:**

Among *n* = 379,049, *n* = 23,035 reported inhaled cannabis use. Inhaled cannabis was associated with asthma overall (adjusted odds ratio (aOR) 1.44, 95% CI 1.26–1.63 for daily use) and among *n* = 221,767 with no lifetime tobacco cigarette use (aOR 1.51 for daily use, 95% CI 1.18–1.93). Inhaled cannabis was associated with chronic obstructive pulmonary disease overall (aOR 1.27 for daily use, 95% CI 1.10–1.46), with a non-significant elevated odds of disease among those with no lifetime tobacco cigarette use (aOR 1.54 for daily use, 95% CI 0.92–2.57).

**Conclusions:**

Inhaled cannabis was associated with asthma and chronic obstructive pulmonary disease after adjusting for tobacco cigarette use. Among those with no lifetime tobacco cigarette use, the association with asthma persisted. Cannabis may be a potential modifiable risk factor for asthma and chronic obstructive pulmonary disease.

**Supplementary Information:**

The online version contains supplementary material available at 10.1007/s11606-025-09833-8.

## INTRODUCTION

Cannabis use has increased in the USA over the last 20 years across all sociodemographic groups^1^ and an estimated 62 million Americans used cannabis in the past year.^2^ More than 75% of adults who use cannabis primarily inhale it,^2^ for example, through smoking, vaporizing, or dabbing.^3^ Cannabis smoke contains thousands of compounds, mostly unidentified, including many of the same compounds in tobacco smoke.^4^ Cannabis is associated with increased airway resistance,^5,6^ impaired gas exchange,^7^ bronchial inflammation,^8,9^ symptoms of cough, sputum production, wheezing, and dyspnea,^10,11^ and chronic bronchitis.^12,13^ As a result, assessing the frequency of inhaled cannabis use over the past 30 days has been proposed as a component of comprehensive clinical care.^14^

Many of these symptoms are hallmarks of chronic respiratory diagnoses; respiratory symptoms plus variable airflow obstruction on spirometry are the diagnostic features of asthma,^15^ whereas chronic obstructive respiratory disease (COPD) is characterized by respiratory symptoms, structural changes in the lungs, and airflow limitation that is not substantially reversible.^16^ However, whether cannabis increases the risks of asthma and chronic obstructive pulmonary disease (COPD) is not yet clear.^10,13,17^ Understanding the impact of inhaled cannabis on asthma and COPD has been challenging due to confounding from tobacco cigarette co-use.^5,10,12,17,22^ Well-powered analyses to isolate the impact of cannabis on lung function independent of tobacco cigarettes have not been possible due to small numbers of adults who use cannabis only without tobacco. Further, cannabis may impact lung function only after decades of prolonged use,^23,24^ and thus, cannabis may appear benign among those who use cannabis lightly,^18^ those who recently initiated cannabis use,^7^ or younger adults.^17,25^ Data from large numbers of individuals at younger and older ages with known frequency of cannabis use—including those with no lifetime tobacco cigarette use—are needed to understand the relationship between inhaled cannabis use and pulmonary health.

To address these knowledge gaps, this study aimed to characterize the relationship between the frequency of past 30 days inhaled cannabis use with asthma and COPD, stratified by age and restricted to adults with no lifetime tobacco cigarette use. We hypothesized that cannabis use would correlate with increased odds of asthma and COPD, independent of tobacco cigarette use, and that associations would differ by age.

## STUDY DESIGN AND METHODS

### Data Source and Sample

We used cross-sectional data from the 2016–2020 Behavioral Risk Factor Surveillance System (BRFSS), an annual telephone survey of a nationally representative sample of community-dwelling adults in the USA. Our sample included adults ages 18–74 years who answered questions regarding cannabis use as detailed in the Supplemental Methods. Respondents who most frequently consumed cannabis via drinking, eating, or an unknown/unspecified way were excluded (2.5%).

### Exposure

Our sample included survey respondents ages 18–74 years who answered the question, “During the past 30 days, on how many days did you use marijuana or hashish?”, excluding those who answered “Don’t know” or refused to answer (< 1%). Among adults who used cannabis in the prior 30 days, we included those who answered the question, “During the past 30 days, which one of the following ways did you use marijuana the most often? Did you usually…” with the option “Smoke it (for example in a joint, bong, pipe, or blunt),” “Vaporize it (for example, in an e-cigarette-like vaporizer or another vaporizing device),” or “Dab it (for example, using waxes or concentrates).”

We quantified inhaled cannabis use as a continuous variable, defined as days of cannabis use in the past 30 days divided by 30. Thus, no cannabis use was scored 0, cannabis use on 15 days out of the past 30 days was scored 0.5 (15/30), and daily cannabis use was scored 1 (30/30). A one-unit change in the cannabis exposure variable quantified the effect of daily cannabis use compared to no cannabis use. Thus, our analysis included all inhaled cannabis users and looked for a dose–response between pulmonary outcomes and days per month of cannabis use.

### Outcomes

Asthma was assessed with the question, “Has a doctor, nurse, or other health professional ever told you that you had asthma?” which was followed with, “Do you still have asthma?” Those with asthma were defined as answering “yes” to both questions. COPD was assessed with the question, “Has a doctor, nurse, or other health professional ever told you that you had chronic obstructive pulmonary disease, COPD, emphysema, or chronic bronchitis?” Those with missing values for these questions (0.2 to 0.6%) were excluded.

### Statistical Analysis

We conducted univariate analyses of the weighted distribution of baseline characteristics by frequency of cannabis use and calculated *p*-values from chi-square (categorical variables) or *t*-tests (continuous variables). We conducted univariate and multivariable logistic regression to examine the association between inhaled cannabis use and each disease outcome. To ascertain whether associations differed by age, we included an interaction term denoting the product between binary age category and cannabis use. The binary age category was dichotomized at age 35 for asthma outcomes as prevalence peaks in childhood and young adulthood^26^ and at age 50 for COPD, as COPD prevalence increases in middle age.^27^ To ascertain whether associations differed by age, we included an interaction term denoting the product between binary age category and cannabis use. All analyses and regression models were weighted to account for the complex survey design using BRFSS weights provided by the U.S. Centers for Disease Control and Prevention. Additional details of the covariates in the statistical analysis are in the online data supplement.

### Sensitivity Analyses

To isolate the association of cannabis inhalation on respiratory health and remove residual confounding due to inhaled tobacco, we conducted two sensitivity analyses: restricting the sample to (1) those with no lifetime tobacco cigarette use, and (2) those with no lifetime tobacco cigarette or e-cigarette use. To examine whether cannabis was associated with any lifetime diagnosis of asthma, we repeated analyses with a supplemental outcome of lifetime asthma, defined as answering “Yes” to ever being diagnosed with asthma by a medical professional and “No” to currently having asthma. Because the prevalence of asthma and COPD exceeds 10% in some populations,^26^ we also conducted a sensitivity analysis converting odds ratios to relative risks using established methods.^28^ This study was based on publicly available de-identified data and was exempt from IRB review. All analyses were done in R version 4.0 (R Core Team, 2020, Vienna, Austria).

## RESULTS

### Characteristics of the Cohort

The cross-sectional study sample consisted of *n* = 379,049 adults 18–74 years who completed the cannabis module over 2016–2020 BRFSS survey years; *n* = 23,035 reported inhaled cannabis use in the prior 30 days (Table 1). Respondents were 49.9% (95% CI 49.6–50.2%) women, 60.8% (95% CI 60.4–61.1%) non-Hispanic White, 61.3% (95% CI 61.0–61.7%) had never smoked tobacco cigarettes, and 62.9% (95% CI 62.6–63.3%) had never used e-cigarettes. Among 221,767 adults with no lifetime tobacco cigarette smoking, *n* = 7277 used cannabis in the last 30 days.

**Table 1:** Baseline demographics by categories of cannabis use among adults ages 18–74 (*n* = 379,049)

	Total (***n*** = 379,049)	No cannabis use (***n*** = 356,014)	Nondaily cannabis use (***n*** = 14,398)	Daily cannabis use (***N*** = 8637)
	Unweighted ***n***, weighted column % (95% CI)	Unweighted ***n***, weighted column % (95% CI)	Unweighted ***n***, weighted column % (95% CI)	Unweighted ***n***, weighted column % (95% CI)
Age				
18–34	66,303, 29.8 (29.4, 30.1)	57,531, 27.8 (27.4, 28.1)	5581, 52.4 (50.8, 54.0)	3191, 50.4 (48.3, 52.4)
35 +	312,746, 70.2 (69.9, 70.6)	298,483, 72.2 (71.9, 72.6)	8817, 47.6 (46.0, 49.2)	5446, 49.6 (47.6, 51.7)
Female sex	200,701, 49.9 (49.6, 50.2)	191,951, 51.1 (50.7, 51.4)	5626, 39.1 (37.5, 40.6)	3124, 33.2 (31.3, 35.2)
Race and ethnicity				
Non-Hispanic White	288,662, 60.8 (60.4, 61.1)	272,311, 60.9 (60.6, 61.3)	10,108, 58.9 (57.3, 60.6)	6043, 59.3 (57.1, 61.4)
Non-Hispanic Black	29,305, 11.6 (11.4, 11.9)	27,140, 11.3 (11.0, 11.5)	1286, 14.8 (13.5, 16.1)	879, 17.2 (15.4, 19.0)
Non-Hispanic Other	33,270, 8.9 (8.7, 9.1)	25,381, 8.9 (8.7, 9.2)	1473, 8.9 (7.9, 9.8)	958, 7.9 (6.9, 9.0)
Hispanic	27,812, 18.7 (18.4, 19.0)	31,182, 18.9 (18.6, 19.2)	1331, 17.4 (16.1, 18.7)	757, 15.7 (14.0, 17.3)
Married	211,062, 51.7 (51/4, 52.1)	204,011, 53.9 (53.6, 54.3)	4532, 27.8 (26.4, 29.2)	2519, 26.8 (25.0, 28.6)
Education				
Less than high school	24,228, 12.4 (12.2, 12.7)	22,267, 12.4 (12.1, 12.7)	1001, 10.7 (9.5, 11.8)	960, 16.9 (15.1, 18.7)
High school degree	100,393, 27.7 (27.4, 28.0)	93,070, 27.3 (27.0, 27.6)	4126, 28.6 (27.1, 30.0)	3197, 37.3 (35.3, 39.3)
Some college	109,172, 31.9 (31.6, 32.2)	101,646, 31.4 (31.1, 31.8)	4741, 39.2 (37.6, 40.7)	2785, 34.1 (32.1, 36.1)
College degree or higher	145,256, 27.9 (27.7, 28.2)	139,031, 28.9 (28.6, 29.2)	4530, 21.6 (20.5, 22.7)	1695, 11.7 (10.7, 12.7)
Annual household income				
< $25,000	77,708, 26.0 (25.6, 26.3)	70,747, 25.4 (25.1, 25.8)	4037, 29.1 (27.6, 30.6)	2924, 35.6 (33.6, 37.6)
$25,000– < 75,000	132,382, 37.3 (36.9, 37.6)	124,191, 37.2 (36.8, 37.6)	4992, 37.1 (35.5, 38.7)	3199, 40.1 (37.9, 42.3)
≥ $75,000	123,875, 36.8 (36.5, 37.1)	118,436, 37.4 (37.0, 37.7)	3876, 33.8 (32.5, 35.4)	1563, 24.3 (22.4, 26.3)
Combustible tobacco cigarette use				
Never	221,767, 61.3 (61.0, 61.7)	214,490, 63.4 (63.1, 63.8)	5324, 44.2 (42.6, 45.7)	1953, 28.6 (26.7, 30.5)
Former	97,820, 22.6 (22.3, 22.8)	91,304, 22.4 (22.1, 22.6)	3958, 23.8 (22.4, 25.1)	2558, 26.8 (25.0, 28.6)
Current	59,462, 16.1 (15.8, 16.3)	50,220, 14.2 (14.0, 14.5)	5116, 32.1 (30.6, 33.6)	4126, 44.6 (42.5, 46.6)
E-cigarette use				
Never	235,895, 62.9 (62.6, 63.3)	229,496, 66.2 (65.9, 66.5)	4241, 27.9 (26.5, 29.4)	2158, 24.2 (22.4, 26.1)
Former	44,260, 14.7 (14.4 14.9)	37,039, 13.0 (12.7, 13.2)	4362, 32.8 (31.2, 34.4)	2859, 35.4 (33.3, 37.4)
Current	11,326, 4.0 (3.9, 4.2)	8,614, 3.2 (3.0, 3.3)	1589, 13.0 (11.8, 14.1)	1123, 14.1 (12.6, 15.5)
Not questioned	66,660, 18.4 (18.2, 18.6)	61,256, 17.7 (17.4, 17.9)	3437, 26.3 (24.9, 27.7)	1967, 26.3 (24.4, 28.3)
Alcohol in past 30 days				
No alcohol use	181,120, 47.6 (47.2, 47.9)	174,465, 49.5 (49.1, 49.8)	3460, 22.9 (21.5, 24.2)	3205, 31.9 (30.0, 33.7)
Non-daily alcohol use	178,394, 48.2 (47.8, 48.5)	164,252, 46.6 (46.2, 47.0)	9657, 70.0 (68.6, 71.5)	4485, 59.0 (57.0, 60.9)
Daily alcohol use	19,535, 4.2 (4.1, 4.4)	17,297, 3.9 (3.8, 4.0)	1291, 7.1 (6.3, 7.9)	947, 9.2 (8.0, 10.3)

Adults who used inhaled cannabis had a lower educational attainment, were more likely to be male, more likely to be non-Hispanic Black, more likely to currently/formerly smoke tobacco cigarettes, and more likely to currently/formerly use e-cigarettes. Daily cannabis use was common among those with chronic lung disease; among those with asthma, 7.4% (95% CI 6.1–8.8%) of those younger than 35 years and 3.1% (95% CI 2.7–3.6%) of those 35 years or older who reported daily use (Table 3). Among respondents with COPD, 10.5% (95% CI 8.6–12.3%) of those younger than 50 years and 2.9% (95% CI 2.5–3.3%) 50 + years reported daily cannabis use.

### Asthma

**Fig. 1:**
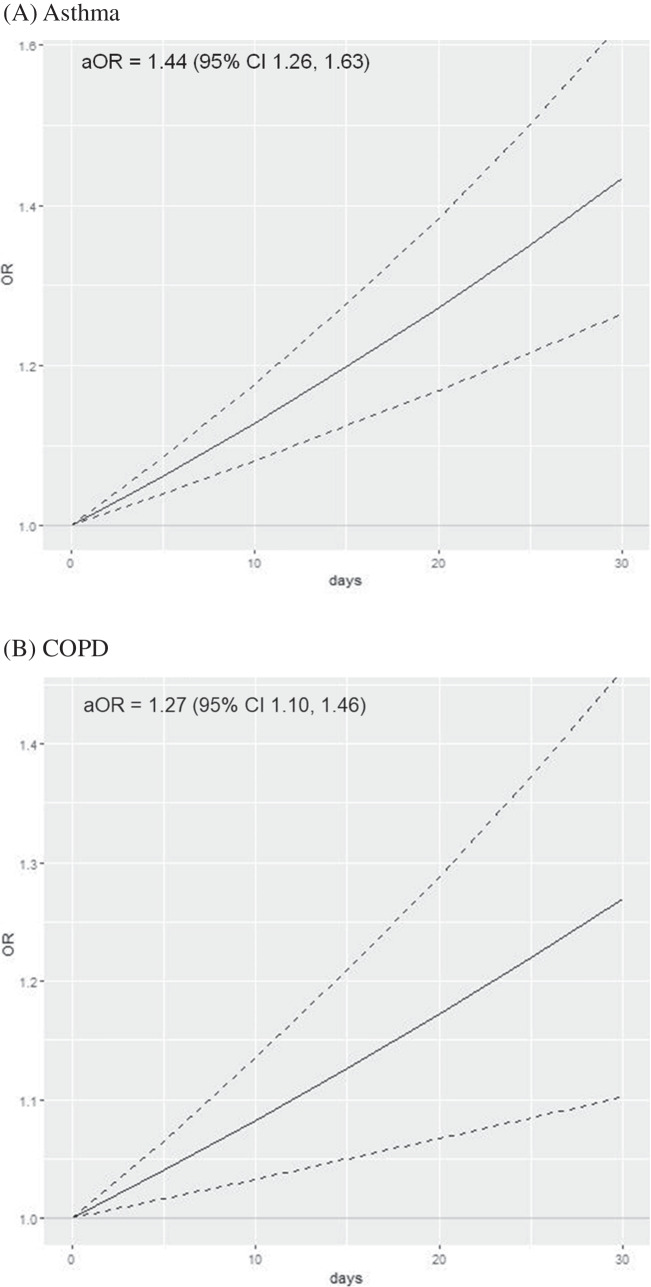
Frequency of cannabis use with asthma and chronic obstructive pulmonary disease among adults ages 18–74 (*n* = 379,049). As frequency of cannabis use increases from 0 to 30 of the past 30 days, the odds of asthma and chronic obstructive pulmonary disease increase significantly. Solid lines denote the adjusted odds ratio (aOR) as frequency of cannabis use increases linearly from non-use (0) to daily use (1) over the past 30 days; dotted lines denote 95% confidence intervals. **A** Asthma. **B** Chronic obstructive pulmonary disease

Among adults who did not use cannabis, asthma prevalence was 8.6% (95% CI 8.1–9.0%) among those < 35 years old and 8.9% (95% CI 8.7–9.2%) among those 35 + (Table 1). The prevalence of asthma increased in each age group as the frequency of past 30-day cannabis use increased; for example, among adults < 35 years, asthma prevalence increased to 12.1% (95% CI 9.9–14.3%) among those who used cannabis daily. Overall, there was a dose–response relationship between days per month of cannabis use and adjusted odds ratios (aOR) of asthma (aOR 1.44, 95% CI 1.26–1.63 for daily use; Fig. 1A) compared to no cannabis use in the prior 30 days. When stratified by age, cannabis use was associated with asthma among adults < 35 years (aOR 1.45, 95% CI 1.19–1.76 for daily use; Table 2) and similarly among adults 35 + years (aOR 1.42, 95% CI 1.21–1.67 for daily use). The magnitude of the cannabis-asthma association did not differ significantly by age (*p* = 0.88 for interaction term).

**Table 2:** Association of cannabis with asthma and chronic obstructive pulmonary disease in adults (*n* = 379,049). The multivariable model included an interaction term denoting the product of age × days of cannabis use per 30 days, to generate age group-specific point estimates

	Asthma	COPD
	aOR (95% CI)^a^	aOR (95% CI)^a^
Days of cannabis use per 30 days^b^		
Younger age group^c^	1.45 (1.19, 1.76)	1.39 (1.13, 1.71)
Older age group^d^	1.42 (1.21, 1.67)	1.13 (0.96, 1.33)
*p*-value for interaction between age and cannabis use^e^	0.88	0.11
Former tobacco smoker	1.16 (1.10, 1.24)	2.82 (2.62, 3.03)
Current smoker	1.35 (1.26, 1.44)	4.85 (4.50, 5.24)

^a^Adjusted for: age, sex, race/ethnicity, body mass index, diabetes, alcohol use (non-use/non-daily use in past 30 days/daily use in past 30 days), educational attainment, physical activity (any physical activity or exercise in the past month/none), marital status (married/unmarried), difficulty paying for medical care (no/yes), and all other variables shown in table^b^Non-use scored as 0/30 = 0, use 15 days/month scored as 15/30 = 0.5, and daily use scored 30/30 = 1. Adjusted odds ratios (aORs) correspond to risk of daily use compared to non-use^c^Younger age group defined as < 35 years for asthma; < 50 years for chronic obstructive pulmonary disease (COPD)^d^Older age group defined as 35 + years for asthma; 50 + years for COPD^e^*p*-value associated with the beta-coefficient of the model term denoting the product of age and cannabis use

#### COPD

The lifetime prevalence of COPD among those who did not use cannabis was 2.9% (95% CI 2.7–3.1%) in adults < 50 years old and 10.1% (95% CI 9.7–10.3%) in adults 50 + years old (Table 1). Cannabis use was associated with an elevated odds of COPD in a dose–response manner overall (aOR 1.27, 95% CI 1.10–1.46 for daily use; Fig. 1B). When stratified by age, cannabis use was associated with COPD among adults < 50 years (aOR 1.39, 95% CI 1.13–1.71 for daily use) but was not significantly associated with COPD among adults 50 + years (aOR 1.13, 95% CI 0.96–1.33 for daily use; Table 2). There was no statistically significant difference in the cannabis-COPD association between older and younger adults (*p* = 0.11 for interaction term).

### Results Among Adults with No Lifetime Tobacco Cigarette Use

**Fig. 2:**
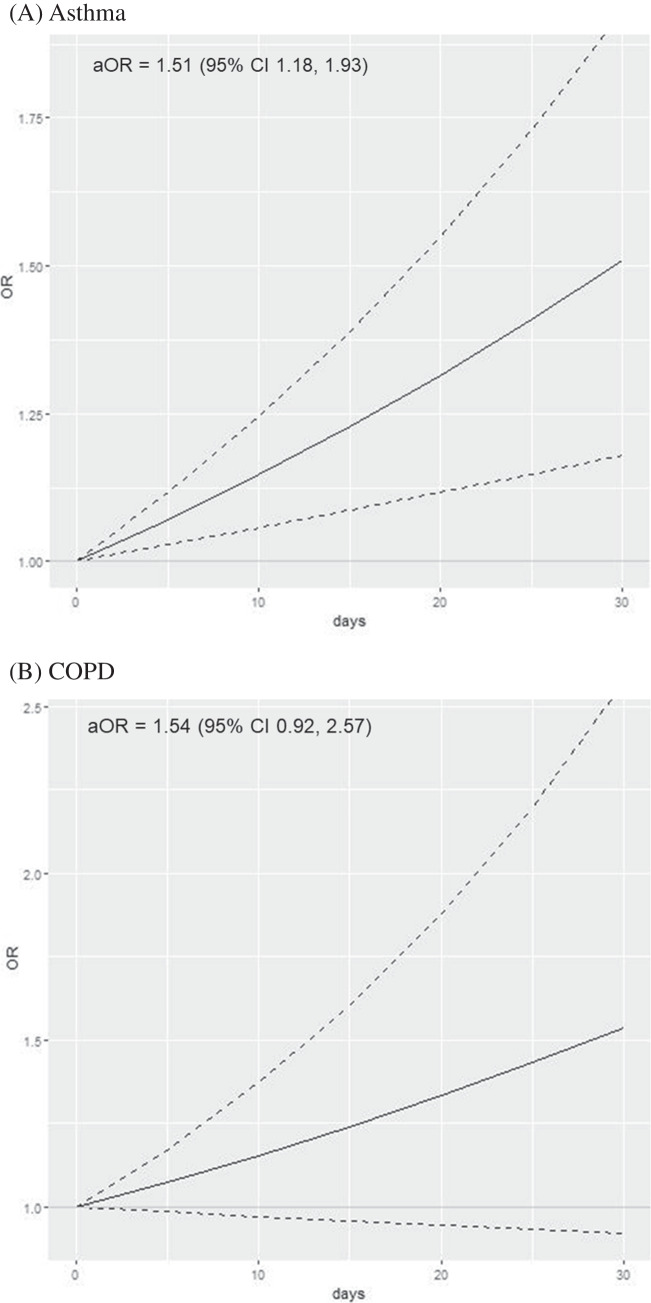
Frequency of cannabis use with asthma and chronic obstructive pulmonary disease among adults ages 18–74 with no lifetime tobacco cigarette use (*n* = 221,767). As frequency of cannabis use increases linearly from 0 to 30 of the past 30 days, the odds of asthma and chronic obstructive pulmonary disease increase. Solid lines denote the adjusted odds ratio (aOR) as frequency of cannabis use increases from non-use (0) to daily use (1) over the past 30 days; dotted lines denote 95% confidence intervals. **A** Asthma. **B** Chronic obstructive pulmonary disease

When analyses were restricted to 221,767 adults with no lifetime tobacco cigarette use, 7277 adults reported inhaled cannabis use in the past 30 days (Table 2). Inhaled cannabis use was associated with asthma (aOR 1.51, 95% CI 1.18–1.93 for daily use; Fig. 2A) in a dose–response manner, compared to those who did not use cannabis. In groups defined by age, daily cannabis use was associated with asthma among those < 35 years (aOR 1.46, 95% CI 1.07–2.00 for daily use; Table 3) and among those 35 + years (aOR 1.60, 95% CI 1.08–2.39 for daily use) to a similar degree (*p* = 0.72 for interaction term). There was no statistically significant association between increasing frequency of cannabis use and COPD overall (aOR 1.54, 95% CI 0.92–2.57 for daily use; Fig. 2B), among adults < 50 years (aOR 1.69, 95% CI 0.88–3.26 for daily use; Table 3) or those 50 + years (aOR 1.20, 95% CI 0.72–1.99). We did not find evidence of significant differences in the associations by age (*p* = 0.42 for interaction term).

**Table 3:** Association of cannabis with asthma and chronic obstructive pulmonary disease in adults with no lifetime tobacco cigarette use (*n* = 221,767). The multivariable model included an interaction term denoting the product of age × days of cannabis use per 30 days, to generate age group-specific point estimates

	Asthma	COPD
	aOR (95% CI)^a^	aOR (95% CI)^a^
Days of cannabis use per 30 days^b^		
Younger age group^c^	1.46 (1.07, 2.00)	1.69 (0.88, 3.26)
Older age group^d^	1.60 (1.08, 2.39)	1.20 (0.72, 1.99)
*p*-value for interaction between age and cannabis use^e^	0.72	0.42

^a^Adjusted for: age, sex, race/ethnicity, body mass index, diabetes, alcohol use (non-use/non-daily use in past 30 days/daily use in past 30 days), educational attainment, physical activity (any physical activity or exercise in the past month/none), marital status (married/unmarried), difficulty paying for medical care (no/yes), and all other variables shown in table^b^Non-use scored as 0/30 = 0, use 15 days/month scored as 15/30 = 0.5, and daily use scored 30/30 = 1. Adjusted odds ratios (aORs) correspond to risk of daily use compared to non-use^c^Younger age group defined as < 35 years for asthma; < 50 years for chronic obstructive pulmonary disease^d^Older age group defined as 35 + years for asthma; 50 + years for chronic obstructive pulmonary disease^e^*p*-value associated with the beta-coefficient of the model term denoting the product of age and cannabis use

### Sensitivity Analyses

When analyses were restricted to 196,520 respondents with no prior tobacco cigarette use or e-cigarette use, cannabis inhalation remained associated with asthma among those < 35 years of age (aOR 1.52, 95% CI 1.01–2.28) and 35 + years of age (aOR 1.73, 95% CI 1.09–2.76; Table E4). There were no significant associations between daily cannabis use and COPD in adults < 50 years (aOR 1.25, 95% CI 0.56–2.81) or adults 50 + years with no tobacco cigarette or e-cigarette use (aOR 1.10, 95% CI 0.63–1.92). There was no meaningful difference in these associations between age groups (*p* = 0.79 for interaction).

The alternative outcome of any lifetime asthma was more prevalent than our primary asthma outcome of current asthma and increased in prevalence as cannabis use increased in frequency (Table E5). Cannabis use was associated with lifetime asthma overall (aOR = 1.51, 95% CI 1.36–1.67) and among those with no lifetime tobacco cigarette use (aOR = 1.50, 95% CI 1.25–1.80; eFigure 1). The association between inhaled cannabis and lifetime asthma persisted in subgroups defined by age, in the overall population and among those with no tobacco cigarette or e-cigarette use (Table E6). There was no evidence of statistically significant differences in these associations by age (*p*-values from 0.36 to 0.79 for interaction terms).

Converting odds ratios to relative risks yielded no meaningful changes to our findings (Tables E7-E9).

## DISCUSSION

In this cross-sectional population-based study of 379,049 adults, inhaled cannabis use was consistently associated with asthma in the overall population, among those with no lifetime tobacco use, across the age spectrum, and in sensitivity analyses using the outcome of any lifetime history of asthma. The odds of asthma increased with increasing frequency of cannabis use in a dose–response manner. Inhaled cannabis use was associated with COPD in the overall population and among adults < 50 years old. We saw an elevated odds of COPD among those with no lifetime tobacco cigarette use in a dose–response manner although this was not statistically significant. These findings demonstrate clear and consistent associations between cannabis inhalation and asthma and raise concern that cannabis inhalation could be associated with COPD.

The high prevalence of cannabis use among those with chronic lung disease is concerning and appears to be increasing in younger birth cohorts. We found a high prevalence of daily cannabis use among younger adults with asthma (9%) or COPD (15%). This highlights the need to screen those with asthma and/or COPD for cannabis use, as part of the comprehensive health evaluation recommended in clinical guidelines for asthma^29^ and COPD.^27^ Assessing cannabis use may be increasingly important in the future as today’s young adults age.

Our results linking cannabis use with asthma are consistent with other work. Analysis of the 2020 National Survey on Drug Use and Health, a population-based survey among those 12 + years in the USA, found associations of nearly identical magnitude between past-30 day cannabis use and asthma.^21^ Past 12-month cannabis use was also associated with asthma medication prescriptions in Norway’s nationalized health records system,^30^ and urine tests positive for cannabis and/or billing codes for cannabis use disorder were associated with asthma in claims data from an integrated US health system.^31^ The present analysis goes beyond these earlier studies^21,30,31^ by finding a similar association among those with no lifetime use of tobacco cigarettes and demonstrates a dose–response relationship between frequency of cannabis use and odds of asthma.

We found an elevated odds of COPD with inhaled cannabis use among adults of all ages and those younger than 50 years. These associations were less precise when we restricted to those with no lifetime tobacco cigarette use to address confounding and were not statistically significant. However, as lack of statistical significance does not provide evidence of no effect,^32^ these results do not provide reassurance that inhaled cannabis is safe with respect to COPD.

This study adds to a growing body of literature that raises concern regarding the association of cannabis with COPD. Cannabis smoke is associated with bronchial inflammation in animal models^33^ and in clinical studies^12^ with similar pathologic findings as in those who smoke tobacco cigarettes.^10,34^ Cannabis is associated with declines in the ratio of forced expiratory volume in one second (FEV1) to forced vital capacity (FVC), which is necessary to diagnose COPD. However, this decline is primarily due to increase in FVC with no consistent detrimental impact on FEV1, a metric of COPD severity.^7,12,17,18,35,36^ FEV1 was examined in one landmark longitudinal study^18^ that included 795 adults who used cannabis only followed over 20 years. The median lifetime cannabis use among participants was relatively light at 0.9 joint-years (1 joint-year being equivalent to 1 joint smoked daily for 1 year); modeling found that cannabis inhalation was associated with declines in FEV1 above > 7 joint-years. In another analysis that included 75 adults who used cannabis only with heavier use (mean 54 joint-years) and 91 adults who used cannabis plus tobacco cigarettes, cannabis use was associated with a decline in FEV1, but results were not reported for those who only used cannabis.^5^ In longitudinal studies, cannabis has been associated with a diagnosis of COPD^18,36^ and precursor changes in pulmonary function^7^ though one study—which included only adults who formerly or currently used tobacco cigarettes—found no impact of cannabis on COPD incidence or progression.^22^

In our analysis, the lack of association between inhaled cannabis and COPD among the age 50 + subgroup (aOR 1.13, 95% CI 0.96–1.33) merits discussion. Today’s older adults may not have accrued sufficient cannabis exposure—in terms of intensity and/or duration—to lead to a measurable association with odds of COPD,^17^ which requires decades to develop fully in contrast to asthma.^37^ In our data, adults older than 50 years had approximately threefold lower prevalence of daily cannabis use than younger adults. Further, the lag time from cannabis initiation to measurable impact on lung function may last decades, but population-level data indicate that older adults have only recently initiated cannabis use. In 2018, the prevalence of cannabis use among those 65 years and older was 5.7%, despite use being essentially zero (0%) in 2002, according to US national surveys.^38^ Any past-year cannabis use among those 50 + years increased from 5.3% in 2015 to 12% in 2021.^39,40^ In a prospective cohort with cannabis use measured as joint-years since age 17, reductions in gas exchange and small airway dysfunction (which may progress to COPD) among adults who used cannabis were only detected after age 45, up to 28 years after initiating cannabis use.^7,41^ In another study, the likelihood of chronic bronchitis increased only after > 10 joint-years of cannabis use.^19^ Thus, it may be that today’s young adults—who use cannabis more heavily than those in the past—may be the first generation to provide sufficient insight into the question of whether cannabis impacts COPD risk as they age. Future analyses with reliable measures of cumulative lifetime cannabis exposure are needed to corroborate our observed association between cannabis inhalation and COPD among younger adults and all adults in aggregate.

This study addressed key limitations of prior research to understand cannabis’ impacts on pulmonary health by analyzing data from a large number of individuals at younger and older ages, reporting results separately for those with no lifetime tobacco cigarette use, and examining associations by frequency of cannabis use. Specifically, our analysis had a large number of adults who inhaled cannabis and denied lifetime tobacco cigarette use, a key group to isolate the health effects of cannabis. A systematic review and meta-analysis on the impact of cannabis on pulmonary function included 22 studies, which in aggregate contributed 243 individuals who used cannabis only.^10^ In contrast, our study included 7277 adults who used only cannabis among 221,767 lifetime tobacco non-smokers. In addition, we restricted to adults who primarily inhaled cannabis, the most biologically plausible route that cannabis would impact pulmonary health, and our exposure of past-30 days inhaled cannabis use matches that proposed for clinical care to screen for cannabis use.^14^

This study has limitations. While cross-sectional studies—including hundreds that rely on BRFSS data^42^—play an essential role in biomedical research to provide preliminary evidence of associations,^43^ including the link between age at initiation of tobacco use and lung cancer risk,^44^ they have specific limitations. First, we could not assess cannabis use prior to each diagnosis. However, an association is biologically plausible^17^ and our results are corroborated by longitudinal studies on COPD^18,36^ or precursor changes in lung function.^7^ Further it is unlikely that the observed dose–response relationships are explained by reverse causality. Second, our outcomes are prevalent diagnoses which tend to be more indolent,^45^ and thus, our results could underestimate associations between inhaled cannabis and aggressive chronic pulmonary disease. Despite these limitations, utilizing cross-sectional data allows us to examine associations that would otherwise require following large prospective cohorts for an extended duration. Answering these questions with a prospective cohort would be resource-intensive, impractical, and unlikely to yield meaningful results quickly. In the meantime, 3.7 million Americans initiate cannabis use each year^2^ and deserve to understand the potential health impacts of cannabis inhalation utilizing the full breadth of information available on the health effects of cannabis use including cross-sectional data. Third, exposure and outcome data were self-reported. However, cannabis is more likely disclosed than other drugs and self-reported use shows good agreement with urine toxicology results.^46^ The majority of our survey data was collected in settings with legal cannabis use which promotes accurate disclosure of use; that is, of 79 state-year combinations in which the module was collected (e.g., Alaska in 2016), cannabis was legal in 49.^47^ Outcomes were self-reported, though COPD^48^ and asthma^49^ may be under-diagnosed. Non-differential misclassification of cannabis use, asthma, or COPD would underestimate associations. Fourth, we do not have an estimate of total lifetime exposure which could introduce exposure misclassification especially for those with heavy cannabis use who recently quit or those who recently initiated use. If those with respiratory conditions were to reduce cannabis inhalation, then this would tend to underestimate associations. Finally, we did not have data on all-known risk factors for chronic lung disease such as breastfeeding, other early childhood exposures, prior pulmonary infections, or occupational exposures,^50^ though the relative contribution of each of these risk factors is overshadowed by that of tobacco smoke^27,51^ which we addressed through covariate adjustment and exclusion those with lifetime tobacco cigarette use.

## INTERPRETATION

This population-based cross-sectional study found that cannabis inhalation was consistently associated with asthma in the overall population, among adults younger and older than 35 years, and among those with no lifetime tobacco cigarette use. Inhaled cannabis was significantly associated with odds of COPD in the overall population and among adults younger than 50 years, but not to a statistically significant degree among adults older than 50 years or those with no lifetime tobacco cigarette use. The magnitude of our associations increased with frequency of cannabis use over the prior 30 days suggesting a dose–response relationship, one component of causal inference. Future research is needed to infer a causal relationship between chronic lung disease and cannabis use, which may be a potential modifiable risk factor in the development of asthma and/or COPD. Clinicians should routinely ask about cannabis use in patients with asthma or COPD and advise that inhaled cannabis is not known to be safe with respect to asthma or COPD.

## Supplementary Information

Below is the link to the electronic supplementary material.

ESM1 (DOCX 164 KB)

## Abbreviations

aOR: Adjusted odds ratio
BRFSS: Behavioral Risk Factor Surveillance System
CI: Confidence interval
COPD: Chronic obstructive pulmonary disease
IRB: Institutional Review Board
OR: Odds ratio

## Declarations:

### Disclaimer:

The study sponsors had no role in the study design; collection, analysis, or interpretation of data; writing the report; or the decision to submit the manuscript for publication. The opinions expressed herein are those of the authors and not their employers, the U.S. Department of Veterans Affairs, the U.S. government, or the study sponsors.
