# Supplementary material for: Inhaled Cannabis, Asthma, and Chronic Obstructive Pulmonary Disease: A Population-Based Cross-Sectional Study of n = 379,049
Source: J Gen Intern Med. 2025 Sep 4;41(4):1077–86. doi: 10.1007/s11606-025-09833-8 (PMC12681257; doi:10.1007/s11606-025-09833-8)
Supplement: Supplementary file 1 — (DOCX 164 KB) [file 11606_2025_9833_MOESM1_ESM.docx]

Supplemental Methods

**Data Source and Sample**

The Behavioral Risk Factor Surveillance System (BRFSS) is an annual survey conducted by the U.S. Centers for Disease Control and Prevention regarding health-related risk factors, chronic conditions and healthcare access.^E1^ Core questions are asked of all respondents; states may opt to add additional topic-specific modules. BRFSS began to include an optional cannabis module in 2016.^E2^ Twenty-seven states and 2 territories contributed data for this analysis; in total, the module was conducted 77 times across 27 states/territories and five years. In 47 of these instances, cannabis was legal in a given state and year.^E3^

**Covariates**

Lifetime tobacco cigarette use was assessed via the following questions. Those with no lifetime tobacco cigarette use answered “No” to the question, “Have you smoked at least 100 cigarettes in your entire life?” From among those who endorsed smoking 100+ lifetime cigarettes, those who formerly used tobacco cigarettes answered “Not at all” to the question, “Do you now smoke cigarettes every day, some days, or not at all?” whereas those who currently used tobacco cigarettes answered “Every day,” or “Some days.” E-cigarette use was assessed with the question, “Have you ever used an e-cigarette or other electronic vaping product, even just one time, in your entire life?” Current and former use of e-cigarettes was identified with the question, “Do you now use e-cigarettes or other electronic vaping products every day, some days, or not at all?” Those currently using tobacco e-cigarettes or other electronic vaping products answered “Every day” or “Some days,” whereas former adults who formerly used e-cigarettes answered “Not at all.”

Aligning with prior analyses of asthma^E4^ and COPD,^E5,6^ adjustment variables included age (per 10 years), sex, self-identified race/ethnicity (non-Hispanic white, non-Hispanic Black, non-Hispanic other, or Hispanic), educational attainment (less than high school, high school, some college, or college graduate), marital status (married/unmarried), difficulty paying for medical care, body mass index (BMI), diabetes, physical activity (any physical activity or exercise in the past month/none), alcohol consumption (non-use, non-daily use, daily use), and smoking tobacco cigarettes (never, former, current).

References Cited in Supplemental Methods

E1. Centers for Disease Control and Prevention. About BRFSS. Accessed October 8, 2021. https://www.cdc.gov/brfss/about/index.htm

E2. Centers for Disease Control and Prevention. CDC - BRFSS - 2016 BRFSS Modules Used by Category. Accessed July 15, 2024. https://www.cdc.gov/brfss/questionnaires/modules/category2016.htm

E3. Jeffers AM, Glantz S, Byers AL, Keyhani S. Association of Cannabis Use With Cardiovascular Outcomes Among US Adults. *J Am Heart Assoc*. Published online February 28, 2024:e030178. doi:10.1161/JAHA.123.030178

E4. Goodwin RD, Zhou C, Silverman KD, Rastogi D, Borrell LN. Cannabis use and the prevalence of current asthma among adolescents and adults in the United States. *Prev Med*. 2024;179:107827. doi:10.1016/j.ypmed.2023.107827

E5. Quach A, Giovannelli J, Chérot-Kornobis N, et al. Prevalence and underdiagnosis of airway obstruction among middle-aged adults in northern France: The ELISABET study 2011-2013. *Respir Med*. 2015;109(12):1553-1561. doi:10.1016/j.rmed.2015.10.012

E6. Salvi SS, Barnes PJ. Chronic obstructive pulmonary disease in non-smokers. *Lancet*. 2009;374(9691):733-743. doi:10.1016/S0140-6736(09)61303-9

**Table E1. Distribution of cannabis use with asthma and COPD among adults, by age (n=379,049).**

| **(A) Younger age group^a^** | | | | |
| --- | --- | --- | --- | --- |
| Asthma | | | | |
|  | No | Yes |  |  |
| Cannabis use | Unweighted n | Unweighted n | Weighted row % (95% CI) | Unadjusted OR^b^ |
| Non-use | 52,031 | 5,047 | 8.6 (8.1, 9.0) | -- |
| Non-daily use | 4,932 | 595 | 10.3 (8.9, 11.8) | 1.24 (1.14, 1.36) |
| Daily use | 2,758 | 395 | 12.1 (9.9, 14.3) | 1.48 (1.32, 1.65) |
| Total | 59,721 | 6,037 | 8.9 (8.5, 9.3) | -- |
| COPD | | | | |
|  | No | Yes |  |  |
| Cannabis use | Unweighted n | Unweighted n | Weighted row % (95% CI) | Unadjusted OR^b^ |
| Non-use | 130,720 | 4,038 | 2.9 (2.7, 3.1) | -- |
| Non-daily use | 8,412 | 376 | 3.7 (2.9, 4.5) | 1.45 (1.30, 1.61) |
| Daily use | 5,081 | 408 | 7.4 (6.0, 8.7) | 2.60 (2.34, 2.89) |
| Total | 144,213 | 4,822 | 3.2 (3.0, 3.3) | -- |
| **(B) Older age group^c^** | | | | |
| Asthma | | | | |
|  | No | Yes |  |  |
| Cannabis use | Unweighted n | Unweighted n | Weighted row % (95% CI) | Unadjusted OR^b^ |
| Non-use | 269,433 | 27,602 | 8.9 (8.7, 9.2) | -- |
| Non-daily use | 7,798 | 964 | 10.7 (9.3, 12.2) | 1.21 (1.13, 1.29) |
| Daily use | 4,675 | 727 | 12.6 (10.7, 14.4) | 1.51 (1.40, 1.64) |
| Total | 281,906 | 29,293 | 9.1 (8.9, 9.3) | -- |
| COPD | | | | |
|  | No | Yes |  |  |
| Cannabis use | Unweighted n | Unweighted n | Weighted row % (95% CI) | Unadjusted OR^b^ |
| Non-use | 197,916 | 22,242 | 10.1 (9.7, 10.3) | -- |
| Non-daily use | 4,668 | 887 | 14.0 (12.3, 15.7) | 1.69 (1.57, 1.82) |
| Daily use | 2,399 | 707 | 17.8 (15.5, 20.0) | 2.62 (2.41, 2.85) |
| Total | 204,983 | 23,836 | 10.3 (10.0, 10.5) | -- |
| ^a^ Younger age group defined as <35 years for asthma; <50 years for COPD  ^b^ Accounts for complex survey sampling  ^c^ Older age group defined as 35+ years for asthma; 50+ years for COPD. | | | | |

**Table E2. Distribution of cannabis with asthma and COPD among adults with no lifetime tobacco cigarette use (n=221,767).**

| **(A) Younger age group^a^** | | | | |
| --- | --- | --- | --- | --- |
| Asthma | | | | |
|  | No | Yes |  |  |
| Cannabis use | Unweighted n | Unweighted n | Weighted row % (95% CI) | Unadjusted OR^b^ |
| Non-use | 36,092 | 6,365 | 14.9 (14.2, 15.4) | -- |
| Non-daily use | 2,471 | 574 | 18.4 (16.0, 20.7) | 1.32 (1.20, 1.45) |
| Daily use | 790 | 212 | 19.0 (15.0, 23.0) | 1.52 (1.30, 1.77) |
| Total | 39,353 | 7,151 | 15.2 (14.6, 15.8) | -- |
|  |  |  |  |  |
| COPD | | | | |
|  | No | Yes |  |  |
| Cannabis use | Unweighted n | Unweighted n | Weighted row % (95% CI) | Unadjusted OR^b^ |
| Non-use | 89,578 | 1,451 | 1.7 (1.5, 1.8) | -- |
| Non-daily use | 3,867 | 59 | 1.4 (0.08, 2.0) | 0.94 (0.72, 1.21) |
| Daily use | 1,384 | 34 | 3.3 (1.2, 5.3) | 1.52 (1.05, 2.10) |
| Total | 94,829 | 1,544 | 1.7 (1.5, 1.8) | -- |
| **(B) Older age group^c^** | | | | |
| Asthma | | | | |
|  | No | Yes |  |  |
| Cannabis use | Unweighted n | Unweighted n | Weighted row % (95% CI) | Unadjusted OR^b^ |
| Non-use | 150,594 | 21,017 | 12.1 (11.8, 12.5) | -- |
| Non-daily use | 1,939 | 324 | 16.0 (13.1, 18.8) | 1.20 (1.06, 1.35) |
| Daily use | 778 | 169 | 17.9 (12.9, 22.8) | 1.56 (1.31, 1.83) |
| Total | 153,311 | 21,510 | 12.2 (11.9, 12.6) | -- |
| COPD | | | | |
|  | No | Yes |  |  |
| Cannabis use | Unweighted n | Unweighted n | Weighted row % (95% CI) | Unadjusted OR^b^ |
| Non-use | 117,820 | 5,152 | 4.4 (4.2, 4.7) | -- |
| Non-daily use | 1,336 | 55 | 3.0 (1.7, 4.4) | 0.94 (0.71, 1.22) |
| Daily use | 479 | 48 | 5.8 (3.1, 8.5) | 2.29 (1.68, 3.05) |
| Total | 119,635 | 5,255 | 4.4 (4.2, 4.7) | -- |
| ^a^ Younger age group defined as <35 years for asthma; <50 years for COPD  ^b^ Accounts for complex survey sampling  ^c^ Older age group defined as 35+ years for asthma; 50+ years for COPD. | | | | |

**Table E3. Frequency of cannabis use by chronic respiratory conditions and age (n=379,049).**

| **(A) Younger age group^a^** | | | |
| --- | --- | --- | --- |
|  | **Lifetime asthma^b^** | | |
|  | No | Yes | p^c^ |
| Cannabis use | weighted column % (95% CI)^d^ | weighted column % (95% CI)^d^ |  |
| Non-use | 86.4 (85.9, 86.9) | 82.0 (80.6, 83.3) | <0.001 |
| Non-daily use | 8.5 (8.1, 8.9) | 10.6 (9.5, 11.6) |  |
| Daily use | 5.1 (4.7, 5.4) | 7.5 (6.5, 8.5) |  |
|  | **Current asthma^e^** | | |
|  | No | Yes | p^c^ |
| Cannabis use | weighted column % (95% CI)^d^ | weighted column % (95% CI)^d^ |  |
| Non-use | 86.0 (85.5, 86.5) | 82.3 (80.5, 84.2) | <0.001 |
| Non-daily use | 8.7 (8.3, 9.1) | 10.3 (8.9, 11.7) |  |
| Daily use | 5.3 (4.9, 5.6) | 7.4 (6.1, 8.8) |  |
|  | **COPD** | | |
|  | No | Yes | p^c^ |
| Cannabis use | weighted column % (95% CI)^d^ | weighted column % (95% CI)^d^ |  |
| Non-use | 85.9 (85.4, 86.4) | 81.7 (79.3, 84.1) | <0.001 |
| Non-daily use | 8.8 (8.4, 9.2) | 7.9 (6.2, 9.5) |  |
| Daily use | 5.3 (4.9, 5.6) | 10.5 (8.6, 12.3) |  |
| **(B) Older age group^f^** | | | |
|  | **Lifetime asthma^b^** | | |
|  | No | Yes | p^c^ |
| Cannabis use | weighted column % (95% CI)^d^ | weighted column % (95% CI)^d^ |  |
| Non-use | 94.6 (94.4, 94.8) | 92.4 (91.8, 93.0) | <0.001 |
| Non-daily use | 3.3 (3.1, 3.4) | 4.2 (3.8, 4.7) |  |
| Daily use | 2.1 (2.0, 2.2) | 3.3 (2.9, 3.8) |  |
|  | **Current asthma^e^** | | |
|  | No | Yes | p^c^ |
| Cannabis use | weighted column % (95% CI)^d^ | weighted column % (95% CI)^d^ |  |
| Non-use | 94.5 (94.3, 94.7) | 92.8 (92.1, 93.6) | <0.001 |
| Non-daily use | 3.3 (3.2, 3.5) | 4.0 (3.4, 4.6) |  |
| Daily use | 2.2 (2.1, 2.3) | 3.1 (2.7, 3.6) |  |
|  | **COPD** | | |
|  | No | Yes | p^c^ |
| Cannabis use | weighted column % (95% CI)^d^ | weighted column % (95% CI)^d^ |  |
| Non-use | 95.7 (95.5, 95.9) | 93.2 (92.6, 93.8) | <0.001 |
| Non-daily use | 2.8 (2.6, 2.9) | 3.9 (3.4, 4.4) |  |
| Cannabis use (continued) | weighted column % (95% CI)^d^ (continued) | weighted column % (95% CI)^d^ (continued) | p^c^ (continued) |
| Daily use | 1.5 (1.4, 1.6) | 2.9 (2.5, 3.3) | <0.001 |
| ^a^ Younger age group defined as < 35 years for lifetime/current asthma; < 50 years for lifetime COPD.  ^b^ Defined as answering “Yes” to ever being diagnosed with asthma by a medical professional and “No” to currently having asthma.  ^c^ p-values from chi-squared tests.  ^d^ Accounts for complex survey sampling  ^e^ Defined as answering “Yes” to ever being diagnosed with asthma by a medical professional and “Yes” to currently having asthma.  ^f^ Older age group defined as 35+ years for asthma; 50+ years for COPD. | | | |

**Table E4. Cannabis and chronic respiratory conditions in adults with no lifetime tobacco cigarette or e-cigarette use (n=196,520).** The multivariable model included an interaction term denoting the product of age × days of cannabis use per 30 days, to generate age group-specific point estimates.

|  | Lifetime asthma | Current asthma | Lifetime COPD |
| --- | --- | --- | --- |
|  | aOR (95% CI)^a^ | aOR (95% CI)^a^ | aOR (95% CI)^a^ |
| **Days of cannabis use per 30 days^b^** | | | |
| Younger age group^c^ | 1.47 (1.08, 2.01) | 1.52 (1.01, 2.28) | 1.25 (0.56, 2.81) |
| Older age group^d^ | 1.83 (1.30, 2.59) | 1.73 (1.09, 2.76) | 1.10 (0.63, 1.92) |
| p-value for interaction between age and cannabis use^e^ | 0.36 | 0.68 | 0.79 |
| ^a^ Adjusted for: age, sex, race/ethnicity, BMI, diabetes, alcohol use (non-use/non-daily use in past 30 days/daily use in past 30 days), educational attainment, physical activity, marital status (married/unmarried) and difficulty paying for medical care (no/yes).  ^b^ Non-use scored as 0/30=0, use 15 days/month scored as 15/30=0.5, and daily use scored 30/30=1. aORs correspond to risk of daily use compared to non-use.  ^c^ Younger age group defined as < 35 years for asthma; < 50 years for COPD.  ^d^ Older age group defined as 35+ years for asthma; 50+ years for COPD.  ^e^ p-value associated with the beta-coefficient of the model term denoting the product of age and cannabis use. | | | |

**Table E5. Distribution of cannabis use and lifetime asthma (n=379,049).**

|  | Lifetime asthma^a^ | |  |  |
| --- | --- | --- | --- | --- |
|  | No | Yes |  |  |
| **(A) Younger age group (<35 years)** | | | | |
| Cannabis use | unweighted n | unweighted n | weighted row % (95% CI)^b^ | Unadjusted odds ratio^b^ |
| Non-use | 48,359 | 9,017 | 15.6 (15.1, 16.2) | NA |
| Non-daily use | 4,480 | 1,079 | 19.6 (17.8, 21.4) | **1.29 (1.20, 1.39)** |
| Daily use | 2,474 | 708 | 22.3 (19.6, 25.0) | **1.53 (1.41, 1.67)** |
| Total | 55,313 | 10,804 | 16.4 (15.9, 16.9) | NA |
| **(B) Older age group (35+ years)** | | | | |
| Cannabis use | unweighted n | unweighted n | weighted row % (95% CI)^b^ | Unadjusted odds ratio^b^ |
| Non-use | 258,933 | 38,964 | 13.1 (12.8, 13.4) | NA |
| Non-daily use | 7,406 | 1,392 | 16.7 (15.0, 18.4) | **1.25 (1.18, 1.32)** |
| Daily use | 4,385 | 1,049 | 19.6 (17.4, 21.9) | **1.59 (1.48, 1.70)** |
| Total | 270,724 | 41,405 | 13.4 (13.1, 13.6) | NA |
| ^a^ Defined as answering “Yes” to ever being diagnosed with asthma by a medical professional and “No” to currently having asthma.  ^b^ Accounts for complex survey sampling. | | | | |

**Table E6. Association of cannabis with lifetime asthma.**

|  | Overall sample (n=379,049) | Restricted to those with no lifetime tobacco cigarette use (n=221,767) |
| --- | --- | --- |
|  | Lifetime asthma^a^ | Lifetime asthma^a^ |
|  | aOR (95% CI)^b^ | aOR (95% CI)^b^ |
| **(A) Younger age group (<35 years)** | | |
| Days of cannabis use per 30 days^c^ | 1.48 (1.28, 1.72) | 1.43 (1.13, 1.79) |
| Former tobacco smoker | 1.16 (1.10, 1.22) | NA |
| Current Smoker | 1.30 (1.231, 1.378) | NA |
| **(B) Older age group (35+ years)** | | |
| Days of cannabis use per 30 days^c^ | 1.53 (1.34, 1.75) | 1.66 (1.24, 2.23) |
| Former tobacco smoker | 1.158 (1.102, 1.216) | NA |
| Current tobacco smoker | 1.302 (1.231, 1.378) | NA |
| **(C) All adults** | | |
| Days of cannabis use per 30 days^c^ | 1.507 (1.363, 1.667) | 1.495 (1.245, 1.797) |
| Former tobacco smoker | 1.158 (1.102, 1.216) | NA |
| Current tobacco smoker | 1.303 (1.231, 1.378) | NA |
| p-value for interaction between age and cannabis use^d^ | 0.75 | NA |
| ^a^ Defined as answering “Yes” to ever being diagnosed with asthma by a medical professional and “No” to currently having asthma.  ^b^ Adjusted for: age, sex, race/ethnicity, BMI, diabetes, alcohol use (non-use/non-daily use in past 30 days/daily use in past 30 days), educational attainment, physical activity, marital status (married/unmarried) and difficulty paying for medical care (no/yes).  ^c^ Non-use scored as 0/30=0, use 15 days/month scored as 15/30=0.5, and daily use scored 30/30=1. aORs correspond to risk of daily use compared to non-use.  ^d^ p-value associated with the beta-coefficient of the model term denoting the product of age and cannabis use. | | |

**Table E7. Risk ratios of cannabis use and chronic respiratory conditions (n=379,049).** The multivariable model included an interaction term denoting the product of age × days of cannabis use per 30 days, to generate age group-specific point estimates.

|  | Lifetime asthma | Current asthma | Lifetime COPD |
| --- | --- | --- | --- |
|  | aRR (95% CI)^a^ | aRR (95% CI)^a^ | aRR (95% CI)^a^ |
| **Days of cannabis use per 30 days^b^** | | | |
| (A) Younger age group^c^ | 1.38 (1.23-1.54) | 1.39 (1.17-1.65) | 1.37 (1.13-1.67) |
| (B) Older age group^d^ | 1.43 (1.28-1.59) | 1.37 (1.19-1.57) | 1.11 (0.96-1.28) |
| ^a^ Adjusted for: cigarette use (current/former/never), age, sex, race/ethnicity, BMI, diabetes, alcohol use (non-use/non-daily use in past 30 days/daily use in past 30 days), educational attainment, physical activity, marital status (married/unmarried) and difficulty paying for medical care (no/yes).  ^b^ Non-use scored as 0/30=0, use 15 days/month scored as 15/30=0.5, and daily use scored 30/30=1. aRRs correspond to risk of daily use compared to non-use.  ^c^ Younger age group defined as < 35 years for asthma; < 50 years for COPD.  ^d^ Older age group defined as 35+ years for asthma; 50+ years for COPD. | | | |

**Table E8. Risk ratios of cannabis use and chronic respiratory conditions in adults with no lifetime tobacco cigarette use (n=221,767).** The multivariable model included an interaction term denoting the product of age × days of cannabis use per 30 days, to generate age group-specific point estimates.

|  | Lifetime asthma | Current asthma | Lifetime COPD |
| --- | --- | --- | --- |
|  | aRR (95% CI)^a^ | aRR (95% CI)^a^ | aRR (95% CI)^a^ |
| **Days of cannabis use per 30 days^b^** | | | |
| (A) Younger age group^c^ | 1.34 (1.11-1.59) | 1.41 (1.07-1.84) | 1.66 (0.88-3.06) |
| (B) Older age group^d^ | 1.53 (1.20-1.92) | 1.52 (1.07-2.12) | 1.18 (0.74-1.81) |
| ^a^ Adjusted for: age, sex, race/ethnicity, BMI, diabetes, alcohol use (non-use/non-daily use in past 30 days/daily use in past 30 days), educational attainment, physical activity, marital status (married/unmarried) and difficulty paying for medical care (no/yes).  ^b^ Non-use scored as 0/30=0, use 15 days/month scored as 15/30=0.5, and daily use scored 30/30=1. aRRs correspond to risk of daily use compared to non-use.  ^c^ Younger age group defined as < 35 years for asthma; < 50 years for COPD.  ^d^ Older age group defined as 35+ years for asthma; 50+ years for COPD. | | | |

**Table E9. Risk ratios of cannabis use and chronic respiratory conditions in adults with no lifetime tobacco cigarette or e-cigarette use (n=196,520).** The multivariable model included an interaction term denoting the product of age × days of cannabis use per 30 days, to generate age group-specific point estimates.

|  | Lifetime asthma | Current asthma | Lifetime COPD |
| --- | --- | --- | --- |
|  | aRR (95% CI)^a^ | aRR (95% CI)^a^ | aRR (95% CI)^a^ |
| **Days of cannabis use per 30 days^b^** | | | |
| (A) Younger age group^c^ | 1.37 (1.06-1.74) | 1.45 (1.01-2.05) | 1.24 (0.57-2.67) |
| (B) Older age group^d^ | 1.65 (1.25-2.14) | 1.63 (1.08-2.39) | 1.09 (0.66-1.76) |
| ^a^ Adjusted for: age, sex, race/ethnicity, BMI, diabetes, alcohol use (non-use/non-daily use in past 30 days/daily use in past 30 days), educational attainment, physical activity, marital status (married/unmarried) and difficulty paying for medical care (no/yes).  ^b^ Non-use scored as 0/30=0, use 15 days/month scored as 15/30=0.5, and daily use scored 30/30=1. aRRs correspond to risk of daily use compared to non-use.  ^c^ Younger age group defined as < 35 years for asthma; < 50 years for COPD.  ^d^ Older age group defined as 35+ years for asthma; 50+ years for COPD. | | | |

**Figure E1.**

**(A) Overall sample (n=379,049).**


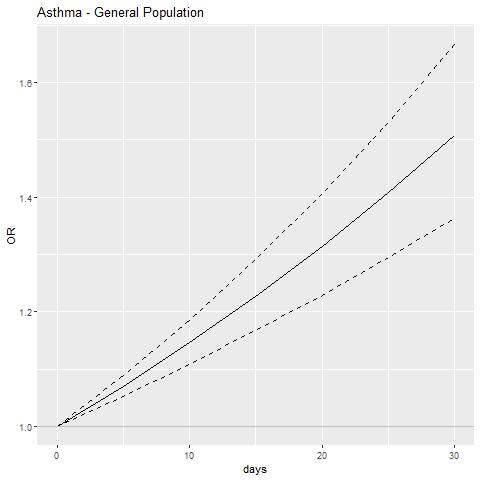


aOR = 1.51 (95% CI 1.36, 1.67)

**(B) Restricted to those with no lifetime tobacco cigarette use (n=221,767).**


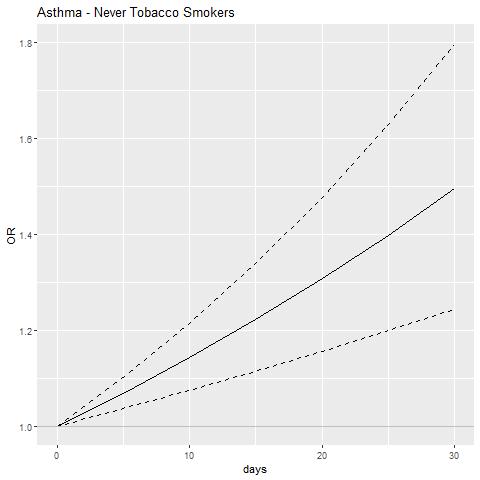


aOR = 1.50 (95% CI 1.25, 1.80)

Supplemental Figure Legends

Figure E1. Frequency of cannabis use and lifetime asthma (n=379,049). As frequency of cannabis use increases from 0 to 30 of the past 30 days, the odds of lifetime asthma increases significantly in (A) the overall sample and (B) those with no lifetime tobacco cigarette use. Solid lines denote the adjusted odds ratio (aOR) as frequency of cannabis use increases from non-use (0) to daily use (1) over the past 30 days; dotted lines denote 95% confidence intervals.
